# An Imaging Overview of COVID-19 ARDS in ICU Patients and Its Complications: A Pictorial Review

**DOI:** 10.3390/diagnostics12040846

**Published:** 2022-03-29

**Authors:** Nicolò Brandi, Federica Ciccarese, Maria Rita Rimondi, Caterina Balacchi, Cecilia Modolon, Camilla Sportoletti, Matteo Renzulli, Francesca Coppola, Rita Golfieri

**Affiliations:** 1 Department of Radiology, IRCCS Azienda Ospedaliero-Universitaria di Bologna, Via Albertoni 15, 40138 Bologna, Italy; ciccarese.f@gmail.com (F.C.); caterinabalacchi@libero.it (C.B.); matteo.renzulli@aosp.bo.it (M.R.); francesca_coppola@hotmail.com (F.C.); rita.golfieri@unibo.it (R.G.); 2 Cardio-Thoracic Radiology Unit, University Hospital S.Orsola-Malpighi, 40138 Bologna, Italy; mariarita.rimondi@aosp.bo.it (M.R.R.); cecilia.modolon@aosp.bo.it (C.M.); csportoletti@hotmail.com (C.S.); 3 Italian Society of Medical and Interventional Radiology, SIRM Foundation, Via della Signora 2, 20122 Milano, Italy

**Keywords:** COVID-19, ARDS, lung CT, mechanical intubation, intensive care, superinfection, pneumothorax, pneumomediastinum, pulmonary embolism

## Abstract

A significant proportion of patients with COVID-19 pneumonia could develop acute respiratory distress syndrome (ARDS), thus requiring mechanical ventilation, and resulting in a high rate of intensive care unit (ICU) admission. Several complications can arise during an ICU stay, from both COVID-19 infection and the respiratory supporting system, including barotraumas (pneumothorax and pneumomediastinum), superimposed pneumonia, coagulation disorders (pulmonary embolism, venous thromboembolism, hemorrhages and acute ischemic stroke), abdominal involvement (acute mesenteric ischemia, pancreatitis and acute kidney injury) and sarcopenia. Imaging plays a pivotal role in the detection and monitoring of ICU complications and is expanding even to prognosis prediction. The present pictorial review describes the clinicopathological and radiological findings of COVID-19 ARDS in ICU patients and discusses the imaging features of complications related to invasive ventilation support, as well as those of COVID-19 itself in this particularly fragile population. Radiologists need to be familiar with COVID-19’s possible extra-pulmonary complications and, through reliable and constant monitoring, guide therapeutic decisions. Moreover, as more research is pursued and the pathophysiology of COVID-19 is increasingly understood, the role of imaging must evolve accordingly, expanding from the diagnosis and subsequent management of patients to prognosis prediction.

## 1. Introduction

Imaging studies performed at the time of admission can help in the risk stratification of patients with COVID-19. In particular, CT findings of COVID-19 pneumonia have been classified by the Radiological Society of North America (RSNA) expert consensus [1] and their variation over a period of time as well as their correlation with patient outcomes has been described by several studies [2,3,4,5].

Some studies have shown that a significant proportion of patients with COVID-19 pneumonia (20–67%) could develop acute respiratory distress syndrome (ARDS), thus requiring mechanical ventilation and resulting in a high rate of intensive care unit (ICU) admission (26–32%) [6,7,8].

According to the American Thoracic Society and the Infectious Diseases Society of America clinical guidelines [9,10], all patients who require invasive mechanical ventilation and/or present with hypotensive shock with the need for vasopressors should be admitted to ICU. Patients should be admitted to ICU also in the presence of at least two minor criteria, i.e., a respiratory rate > 30 respirations/minute, PaO_2_/FiO_2_ < 250, multi-lobar infiltrates, confusion or disorientation, uremia (BUN > 20 mg/dL), leukopenia, thrombocytopenia (platelets < 100,000 cm/mm^3^), hypothermia (temperature < 98.2 °F), and hypotension requiring aggressive fluid administration [11].

Several studies reported that COVID-19 patients who have been admitted to ICU had more comorbidities and, interestingly, were more frequently males, suggesting a sexual dimorphism in the innate response to infections [12].

Low flow nasal oxygen (<6 L/min), high flow nasal oxygen (10–20 L/min), continuous positive airway pressure (CPAP), and bilevel positive airway pressure (BPAP) are some of the non-invasive techniques used in patients with COVID-19 ARDS to improve blood oxygenation while maintaining appropriate SpO_2_ levels [13,14].

Moreover, several studies have demonstrated the benefit of putting patients with COVID-19 with hypoxemia into the prone position [15,16,17]. In fact, since the posterior surface of the lungs is larger than the anterior surface, the prone position allows to minimize the de-recruitment on this large surface area, thus improving lung oxygenation [18].

Intubation and mechanical ventilation should be considered in the case of rapid progression of hypoxemia and no improvement after high flow oxygen administration, worsening mental status, hypercapnia, hemodynamic instability or multiorgan failure, and is required in most patients with ARDS [19,20]. Recently, it has been demonstrated that admission computed tomography (CT) scans of COVID-19 patients can effectively and reliably be used to determine risk factors for invasive ventilation in ICU [21]. In fact, critically ill patients that required invasive ventilation exhibited more imaging findings associated with the later stages of the disease and superimposed findings, such as subpleural band-like consolidations predominantly in dorsal lower lobes. The decision to intubate patients with COVID-19 has been controversial, with some authors recommending early intubation and others trying a stepwise approach to avoid intubation, if possible [22]. This variation is largely due to the evidence that patients on mechanical ventilation are prone to complications, such as barotrauma with alveolar rupture and superimposed bacterial pneumonia, that can often be identified simultaneously on the same patient [23,24].

When a patient continues to have refractory hypoxemia despite optimal mechanical ventilator and medical therapy, extracorporeal membrane oxygenation (ECMO) should be considered for those without contraindications [25,26]. ECMO can bypass the lungs through a veno-venous circuit (VV-ECMO), providing quite complete respiratory support. In particular, the VV-ECMO circuit drains venous blood through a cannula commonly positioned in the inferior vena cava, oxygenates the blood with the external membrane oxygenator, and then pumps the blood back into the same venous compartment, generally through a return cannula positioned in the superior vena cava [27]. The use of veno-arterial ECMO (VA-ECMO) in COVID-19 patients is reserved for those with severe respiratory failure accompanied by severe heart failure or other causes of cardiogenic shock.

Several complications can arise during an ICU stay, from both COVID-19 infection and the respiratory supporting system, including barotraumas (pneumothorax and pneumomediastinum), superimposed pneumonia, coagulation disorders (pulmonary embolism, venous thromboembolism, hemorrhages and acute ischemic stroke) and abdominal involvement (acute mesenteric ischemia, pancreatitis and acute kidney injury) [11,28,29,30]. Therefore, the management of critically ill COVID-19 patients is complex, involving multiple interventions and requiring numerous and potentially interactive therapies that are delivered by a variety of healthcare professionals.

Imaging plays a pivotal role in the detection and monitoring of ICU complications, most of which are life-threatening, thus improving patient outcomes and overall survival. Moreover, proper and correct identification of COVID-19 complications in ICU patients is fundamental, since the comorbidities of this severely ill population may camouflage typical clinical and radiological findings, thus possibly delaying the appropriate treatment [31].

Conventional X-ray is the first-line imaging modality used to evaluate tube positions in the ICU and complications associated with mechanical ventilation. CT represents the best modality choice to indicate vascular alterations, both pulmonary and visceral and can provide adequate information on the state of the gastrointestinal and renal systems, also allowing for the evaluation of possible brain injuries.

The present pictorial review will describe the clinicopathological and radiological findings of COVID-19 ARDS in ICU patients and will discuss the imaging features of complications related to mechanical ventilation and ECMO, as well as those of COVID-19 itself in this particularly fragile population.

## 2. COVID-19 ARDS

COVID-19 ARDS is diagnosed when a patient with confirmed COVID-19 infection develops acute respiratory distress and meets the Berlin 2012 ARDS diagnostic criteria which include: (i) acute hypoxemic respiratory failure (ii) developing within 1 week of onset of symptoms (iii) with bilateral air space opacities on X-ray or CT (iv) not explained by cardiac failure or fluid overload [32,33].

The imaging features of COVID-19 ARDS are similar to ARDS from other etiologies and consist of a rapid progression of lung opacities from unilateral to multifocal or bilateral involvement, with predominantly peripheral location, lymphadenopathy and pleural effusion [34,35,36]. In the acute phase, there is a predilection for dense consolidation to involve the dependent posterior lower lobes with relative sparing of the anterior or non-dependent areas [37]. This evidence represents the rationale behind the implementation of a prone position for ARDS patients. It is important to be aware of when ICU patients are imaged in this position, as they may demonstrate an apparent improvement in consolidations in imaging [38].

However, many differences between COVID-19 ARDS and ARDS caused by other conditions have emerged. First of all, COVID-19 ARDS has a slight delay in onset ranging from 8 to 12 days compared to non-COVID-19 ARDS from other causes where the onset is within 7 days [39]. Moreover, a discrepancy in lung compliance has been supposed, with a possible dissociation between relatively well-preserved lung mechanics and the severity of hypoxemia [40,41]. A possible explanation for severe hypoxemia occurring in compliant lungs could be attributed to the loss of lung perfusion regulation and the consequent hypoxic vasoconstriction. In fact, patients with COVID-19 ARDS develop significantly more thrombotic complications as compared to patients with non-COVID-19 ARDS [42,43,44], thus leading to worse clinical outcomes due to both pulmonary and extra-pulmonary complications [45,46].

Pulmonary fibrosis is a recognized sequela of ARDS caused by other viruses. In severe acute respiratory syndrome (SARS), reticular changes were seen in half of the patients after 4 weeks, but the 15-year follow-up demonstrated that the interstitial abnormalities recovered over the first 2 years, with only 4.6% of patients still having interstitial abnormalities at 15 years [47]. In Middle East respiratory syndrome (MERS), chest X-ray abnormalities consistent with fibrosis were reported in 33% of patients at a median of 43 days from recovery [48] while, in H1N1 influenza, pulmonary fibrosis was recorded in 40% of patients who developed ARDS [49].

**Figure 1:**
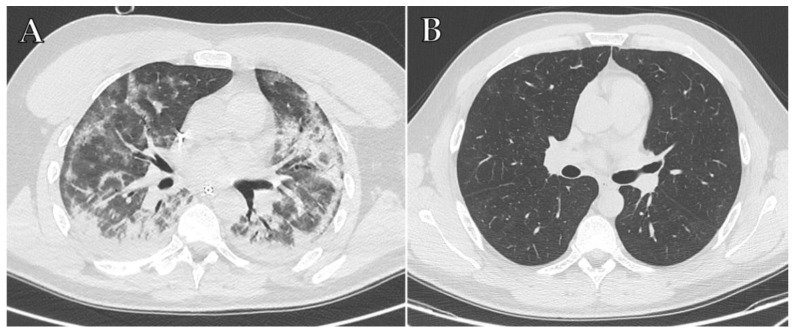
Axial HRCT images of a 38-year-old man with COVID-19 ARDS admitted to ICU at the same level, performed at different times: baseline scan (**A**) and 7-month follow-up (**B**). The baseline scan (**A**) shows typical imaging features indicative of severe COVID-19 pneumonia, including extensive bilateral parenchymal consolidations, mainly affecting the posterior regions of lower lobes, bilateral focal ground-glass opacities in the anterior regions and patchy consolidation, peripherally distributed, resembling pulmonary fibrosis. The 7-month scan (**B**) shows a complete resolution of the parenchymal consolidations and the apparent fibrotic abnormalities.

However, the temporal evolution of COVID-19 ARDS imaging features is still unknown, and the majority of radiological studies involved a small number of patients or still have a short follow-up to complete, thus its long-term sequelae are yet to be identified [37,50]. A 3-month follow-up CT study [51] demonstrated that reticular or fibrotic lesions were present in approximately 50% of patients who were admitted to ICU for COVID-19 ARDS; moreover, these findings were higher compared to that of residual radiographic survivors with other viral pneumonias. However, a more recent study [52] reported that only approximately 14% of COVID-19 patients discharged from ICU kept fibrotic-like changes at the 1-year follow-up, thus suggesting a progressive regression of residual lung damage and, moreover, the residual lung damage rate was slightly lower compared to other causes of ARDS (Figure 1A,B). Therefore, the risk of developing long-term pulmonary sequelae after COVID-19 pneumonia is still a matter of debate and requires further investigations due to the lack of longer follow-ups for this relatively novel disease.

## 3. Pneumothorax

Pneumothorax represents the most common form of barotrauma [53] and develops whenever alveolar pressure increases, resulting in the rupture of alveoli, air dissection along the pulmonary interstitium and eventually air spreading into the pleural space (Macklin effect) [54].

The incidence of pneumothorax in ICU patients with COVID-19 is higher than what is reported for other ICU patients, ranging from 4% to 15%, regardless of the ventilatory strategies [55,56,57]. Moreover, its frequency further increased in the case of prolonged mechanical ventilation or refractory respiratory insufficiency requiring ECMO [58,59]. The occurrence of pneumothorax in ICU patients with COVID-19 has been associated with increased overall mortality [60], therefore, its prompt and correct detection is fundamental.

Pneumothorax is usually secondary to mechanical ventilation with high positive end-expiratory pressure (PEEP) or the cannulation of subclavian or neck vessels [23]. However, several studies [61] have interestingly pointed out that about 33–38% of air leaks occurred in COVID-19 patients who never received ventilatory support, raising the question of whether these patients are more prone to develop air leaks than patients with ARDS for other causes.

**Figure 2:**
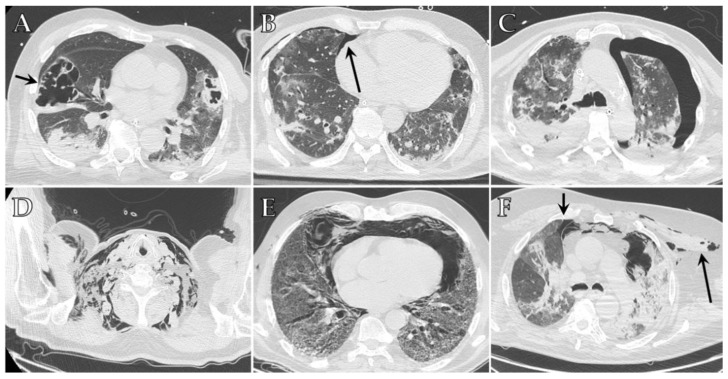
Axial HRCT images of different patients with COVID-19 ARDS admitted to ICU showing imaging features indicative of barotrauma secondary to mechanical ventilation: (**A**) large pneumatocele (black arrow); (**B**) small anterior pneumothorax (black arrow); (**C**) large left pneumothorax; (**D**) extensive bilateral subcutaneous emphysema of the neck; (**E**) pneumomediastinum; (**F**) pneumomediastinum associated with small anterior pneumothorax (small black arrow) and subcutaneous emphysema of the left upper chest (long black arrow).

With this regard, it has been speculated that the air-filled cysts developing in the diseased areas of the lungs as a late consequence of ARDS, especially those peripherally located, may progress to pneumatocele and then spontaneously rupture, thus explaining the significant number of air leaks occurring in patients who had never received ventilatory support [56] (Figure 2A). Therefore, baseline CT imaging may be predictive for patients who may develop pneumothorax [61].

Chest X-ray is an easy-to-use imaging technique to evaluate patients on ventilators but, despite its high specificity (99%), has a low sensibility for diagnosing pneumothorax (38%) [38].

Pneumothorax classically appears as a thin opaque line representing the visceral pleura outlined by lucent air on both sides, corresponding to the air in the pleural space on the chest wall side and in the lung parenchyma on the hilar side, respectively; other signs include the absence of distal lung markings and hypertranslucency of the pleural space [62]. On the standard upright view, air typically starts to collect in the apical region and laterally more than medially. However, in most ICU patients with suspected barotrauma, only supine X-rays are possible, thus pneumothorax becomes more difficult to detect [63]. In this position, the air is usually most visible at the anteromedial or subpulmonary location, outlines the structures of the mediastinum and may enlarge the costophrenic angle (the deep sulcus sign) [38].

Chest CT can easily identify pneumothorax on lung windows, though this imaging technique is not routinely recommended as the first diagnostic modality and is generally used as confirmatory for doubtful cases [64] (Figure 2B,C). Recently, Palumbo et al. [65] have demonstrated that early detection of the Macklin effect in CT scans could help to identify those patients at high risk for clinically relevant barotrauma, and therefore can benefit from different ventilation applications.

## 4. Pneumomediastinum, Pneumopericardium and Subcutaneous Emphysema

Pneumomediastinum has been identified as the second most common barotrauma-related event in patients with mechanical ventilation for COVID-19 ARDS [54]. The mechanism of development of pneumomediastinum is the same as pneumothorax and is linked to the Macklin phenomenon, so much so that the association between these two entities has been widely reported [66]. It can be either spontaneous or secondary to mechanical ventilation in COVID-19 patients and occurs when air leaks from ruptured alveoli into the mediastinum [62]. Rarely, the released air can track to the subcutaneous tissue, causing subcutaneous emphysema (Figure 2D), and/or to the pericardial tissue causing pneumopericardium [54,67,68].

The occurrence of pneumomediastinum in patients with COVID-19 ARDS is higher compared to patients who had ARDS due to other causes, despite protective mechanical ventilation strategies [69], and is associated with a poorer outcome and an increased mortality rate [70].

Pneumomediastinum can be identified on chest X-ray by radiolucent air surrounding the normal anatomic structures within the mediastinum, well demarcating the great vessel and the cardiac contour, especially along its left border. When air dissects inferiorly into the infracardiac region, it separates the cardiac and diaphragmatic densities, and the diaphragm can be seen in its entire extent [71]. When multiple vertically oriented lucencies are visualized extending superiorly into the neck, subcutaneous emphysema must be suspected [72]. Isolated pneumopericardium rarely occurs and manifests as a single band of gas that outlines the pericardium, especially along the left ventricle and right atrium contour [38].

On chest CT, pneumomediastinum is visualized as gas outlining the structures within the mediastinum (Figure 2E,F). Air collection in the extrapleural space may resemble pneumothorax, but the presence of thin connective tissue webs outside of the pleural line is typical of pneumomediastinum and can help achieve a correct differential diagnosis [38]. Chest CT is performed to confirm the diagnosis in equivocal cases on chest X-ray, to assess the extent of the pneumomediastinum, and to evaluate the presence of pneumopericardium, which may have a less favorable prognosis and might require treatment [73].

## 5. Ventilator-Associated Pneumonia (VAP) and Invasive Pulmonary Aspergillosis (IPA)

VAP is the most common ICU-acquired infection and is defined as an infection of pulmonary parenchyma developed in patients receiving mechanical ventilation for at least 48 h [74]. Previous reports indicated that COVID-19 patients have an increased risk of VAP compared to other ARDS, with a variable incidence of 29–79% (vs. 13%), and present even higher mortality rates [75,76]. The increased risk of VAP in ICU patients with COVID-19 is suspected to be due to multiple factors, including prolonged duration of mechanical ventilation, the extensive use of prone positioning, a higher risk for pulmonary infarction, disease, and therapy-associated immune impairment, possibly further amplified by ICU overcrowding during the pandemic outbreak [75].

The most common causative pathogens of VAP are bacteria, which can colonize in the endotracheal/tracheostomy tube of ICU patients. Several studies have demonstrated that the distribution of infecting organisms is similar between patients with and without COVID-19, with Enterobacteria (mainly *Escherichia coli* and *Klebsiella pneumoniae*) representing the most frequent pathogens (in about 40% of cases), followed by other Gram-negative bacilli (such as *Pseudomonas aeruginosa*) and Gram-positive cocci (such as *Staphylococcus aureus* and *Streptococcus pneumoniae*), both accounting for about another 25% of cases each [77,78,79].

Diagnosis of VAP is challenging in COVID-19 patients, due to the overlap of VAP imaging features with those of worsening COVID-19 pneumonia, and laboratory results provide essential support to both clinicians and radiologists [80]. Generally, a sudden increase in X-ray opacities should raise suspicion for VAP, especially if paired with rapid clinical deterioration of the patient [81].

**Figure 3:**
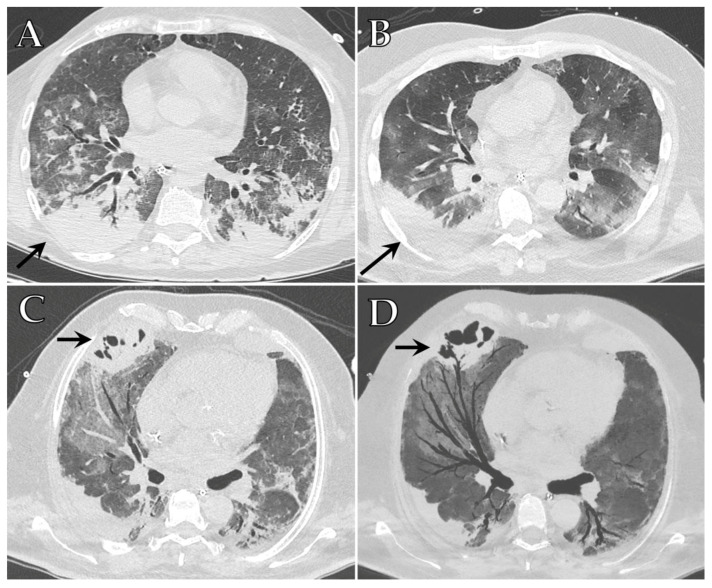
Axial HRCT images of different patients admitted to ICU demonstrating a sudden increase of large consolidations (black arrows) (**A**,**B**) associated with worsening of COVID-19 pneumonia. Superimposed infections of *Acinetobacter* (**A**) and *Aspergillus* (**B**) were detected. A different COVID-19 patient showed pulmonary consolidation complicated by cavitation located in the medium lobe (black arrow) (**C**), which demonstrates communication with the bronchial tree at minimum intensity projection (MIP) reconstruction (black arrow) (**D**); superinfection of *Pseudomonas aeruginosa* was detected.

Chest CT may show imaging features atypical for COVID-19 like lobar consolidation, pleural effusion, mediastinal lymphadenopathy and cavitation, which should prompt the radiologist to consider a bacterial co-infection [82] (Figure 3A,C,D).

Recently, IA secondary to *Aspergillus* invasion has been recognized as a severe complication of COVID-19 infection in ICU patients, with a higher mortality rate (up to 50%) and increased incidence (18–39% vs. 7% of patients) compared to ICU patients with other causes of ARDS [83,84]. Therefore, it is vital to raise awareness among radiologists and clinicians regarding fungal co-infection in COVID-19 patients, due to their immune dysregulation and wide administration of corticosteroids [85,86].

Chest CT is the imaging modality of choice to diagnose IA, as it can detect pulmonary nodules with surrounding ground-glass infiltrates (halo sign), which reflect angio-invasion and hemorrhage in the area surrounding the fungal infection. Sometimes these nodules may cavitate and a fungal ball can be seen within the cavity, with an air-crescent sign [87,88] (Figure 3B).

Although frequently secondary to bacterial, fungal, or mycobacterial co-infections [89,90,91], lung cavitation has also been reported as an atypical imaging feature of late stages of COVID-19 pneumonia [92]. In particular, COVID-19 cavities may be related to barotraumas, pulmonary embolism and infarction with a subsequent necrotic lesion of the lung tissue, or simply to necrotic evolution of the denser consolidations, as well as to all these factors working together [93].

Mechanically ventilated patients are also at increased risk of aspiration pneumonia (AP).

Worsening of X-ray opacities or the appearance of new ones, especially in the retrocardiac and infrahilar regions, should raise the suspicion of AP. Confirmation chest CT may demonstrate the presence of tree-in-bud, centrilobular nodules or consolidations in the dependent parts of the lungs, usually most pronounced in the lower lobes in supine patients or in the upper lobes and right middle lobe in prone patients [38].

## 6. Vascular Complications

The high prevalence of the thrombotic events in patients with COVID-19 may be induced by cross-talking between immune and coagulation systems since the high levels of inflammatory cytokines (such as IL-6 and TNF) induce activation of the endothelial cells and tissue factor, which triggers the coagulation cascade. Accordingly, D-dimer was reported as the most prominent factor in determining COVID-19 severity and subsequent complications [11].

COVID-19 patients in ICU showed a significant increase in the cumulative incidence of symptomatic venous thromboembolism compared with those not requiring ICU care [94], ranging between 31% and 69% [29,95]. Similarly, the percentage of COVID-19 ICU patients that were diagnosed with pulmonary embolism (PE) was higher compared to non-COVID-19 patients, despite having a similar severity to ARDS (23–47% vs. 7.5%) [42,95,96,97,98]. Moreover, thromboembolic complications have been described even in patients treated with anticoagulation therapy from admission, highlighting the intrinsic thrombogenicity of COVID-19 [95]. Due to prolonged immobility, mechanical ventilation and the use of sedatives and neuromuscular blockers, ICU patients experience a significant flow stasis, which could explain their increased risk for thromboembolic complications. Moreover, the frequent insertion of central venous catheters may cause vessel injury, which can further precipitate thrombotic risk in these vulnerable patients [99]. Finally, ECMO may significantly perturb the normal balance of hemostasis with biomaterial-mediated activation of coagulation, complement and inflammatory cascades, as well as increased platelet activation [100,101].

**Figure 4:**
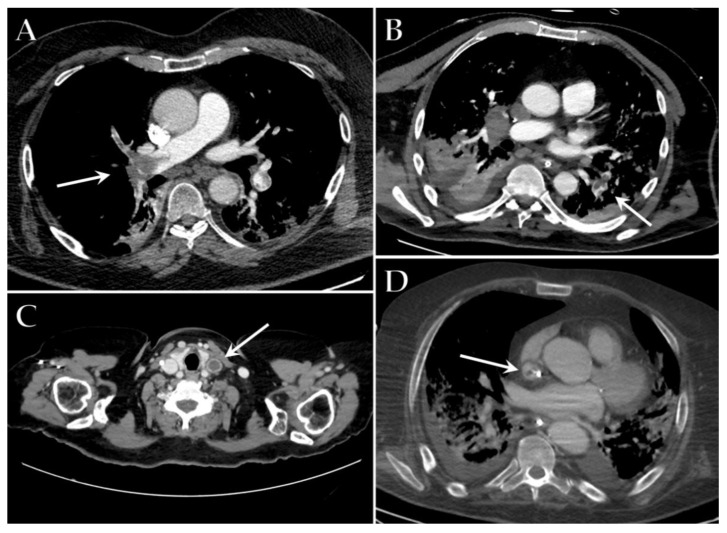
Axial contrast-enhanced CT images of different ICU patients with COVID-19 show filling defects indicative of a massive lung embolism in the right pulmonary artery (white arrow) (**A**), multiple embolisms in the left lower lobe artery (white arrow) (**B**), thrombosis of the left jugular vein (white arrow) (**C**) and thrombosis of the superior vena cava near the central venous catheter (white arrow) (**D**).

The imaging features of venous thrombosis in COVID-19 patients appear analogous to those associated with other diseases (Figure 4C,D). However, radiologists need to be familiar with the high prevalence in these patients, to achieve early detection and a more favorable outcome. The presence of a filling defect within a vessel with hypodense thrombus, venous distension, rim enhancement of the vein, and perivenous edema or fat stranding are the characteristic findings of venous thrombosis. Patients with a requirement for long-term vascular access may develop catheter-related thrombosis, especially when devices are in peripheral veins and in the context of hemodialysis [102].

In COVID-19 patients, PE is more commonly found in the segmental and lobar branches rather than in the central pulmonary arteries and is not confined to areas of the diseased lung, suggesting a diffuse vascular process [103] (Figure 4A,B). Moreover, vascular enlargement has been frequently described in COVID-19 pneumonia, possibly explaining the reason why many COVID-19 patients with PE only have mild symptoms or are even asymptomatic, since it might mitigate the increase of resistance in the pulmonary circulation [99].

Postprocessing vascular mapping using 3D MIP and volume rendering can be used to detect the filling defect more easily.

Besides thromboembolic complications, ICU patients with COVID-19 may also develop hemorrhagic events, with a higher incidence compared to patients on floor units (7.6–8% vs. 1.9%) [104].

Hemorrhages are more frequently observed in patients receiving therapeutic anticoagulation for the prevention of venous thromboembolism [105], but both high levels of proinflammatory cytokines and widespread endothelial cell damage may contribute to their occurrence [106]. Moreover, anticoagulant therapy to prevent the ECMO circuit from clotting and consequently minimize the risk of thromboembolic events may lead to an increased risk for spontaneous hemorrhages anywhere in the body [27].

**Figure 5:**
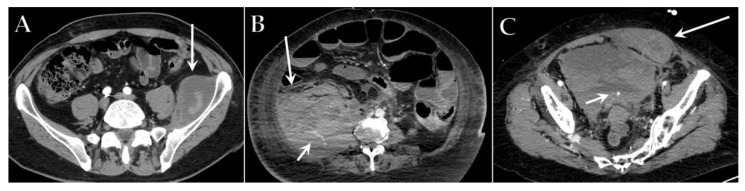
Axial contrast-enhanced CT images of different patients with COVID-19 admitted to ICU that developed hemorrhagic events: (**A**) a hematoma of the left iliopsoas muscle (white arrow); (**B**) a large retroperitoneal hematoma (long white arrow) that displaces the small bowel and shows a blush of active arterial bleeding (small white arrow); (**C**) a spontaneous hematoma of the anterior rectus abdominus (long white arrow) collecting in the pelvis that shows a small blush of active arterial bleeding (small white arrow).

Hematomas appear as variable size hyperdense masses and can cause displacement and compression of surrounding structures (Figure 5A,B). A subacute hematoma might present a blood-fluid hematocrit level due to the evolution of blood products accumulated over time. Contrast-enhanced CT may provide confirmatory evidence of active bleeding, presenting as a blush in the arterial phase (Figure 5B,C).

Iatrogenic hemorrhages at the cannulation or surgical site reached an incidence of 26–31%, but the upper aerodigestive tract (including nose and mouth), the gastrointestinal tract, the genitourinary tract, and the thoracic and abdominal cavities are other relatively common sites of bleeding. The most common muscular site of hematoma is the iliopsoas involving the retroperitoneum, followed by the rectus sheath involving the anterior abdominal wall [104,107,108,109].

## 7. Gastrointestinal Complications

Intestinal involvement related to COVID-19 results from either a direct viral infection, virus-induced inflammatory response, or bowel wall ischemia due to disease hypercoagulability [110,111]. Mesenteric ischemia, ileus, colitis and terminal ileitis are the most common gastrointestinal complications.

The prevalence of gastrointestinal symptoms and complications in ICU patients with severe COVID-19 is higher compared with those with mild disease (16.6% vs. 11.7%, respectively) [44,112,113,114] and with ICU patients without COVID-19 [115].

**Figure 6:**
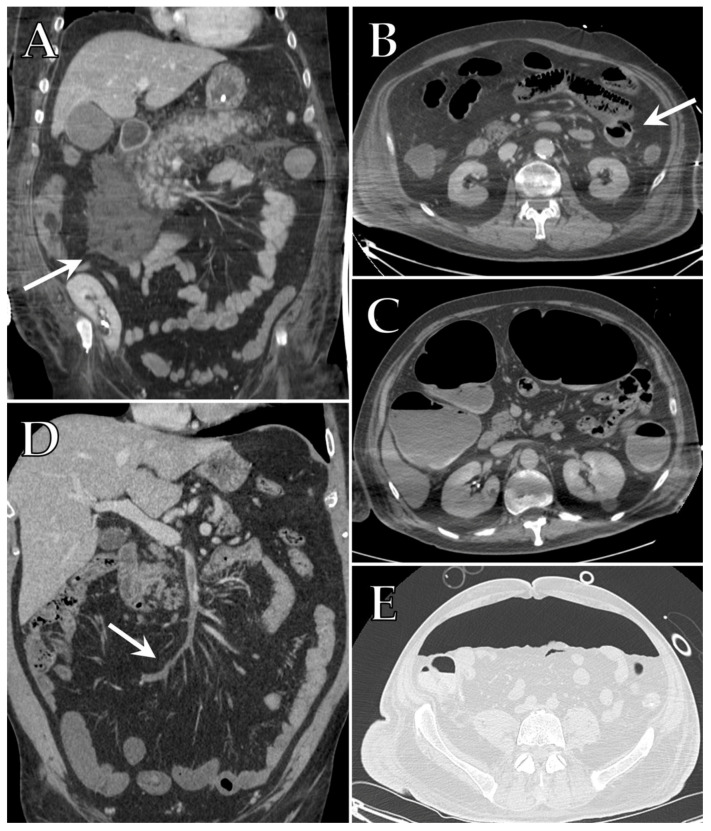
Coronal contrast-enhanced CT image of a patient with COVID-19 admitted to ICU with suspected pancreatitis reveals a diffusely enlarged pancreas, with ill-defined borders, stranding of the peri-pancreatic fat and streak of fluid along the anterior conal fascia (white arrow) (**A**). Axial contrast-enhanced CT scan of different COVID-19 patients with suspected acute mesenteric ischemia demonstrating the presence of linear collections of gas inside the small bowel wall referable to pneumatosis intestinalis (white arrow) (**B**) and dilated intestinal loops with a paper-thin wall and air-fluid levels (**C**). Coronal contrast-enhanced CT image of a COVID-19 patient admitted to ICU showing thrombosis of the superior mesenteric vein extending to several jejuno-ileal branches (white arrow), with consequent loss of contrast enhancement of the corresponding vascularized loops (**D**). Axial unenhanced CT scan with lung window of an ICU patient with COVID-19 and abdominal pain, showing a large quantity of free air (**E**).

CT represents the gold standard for definitive diagnosis of acute mesenteric ischemia, since the US may be limited in obese patients and in the case of pneumoperitoneum or an excessive amount of bowel gas. Acute mesenteric ischemia appears as a filling defect within the lumen of the abdominal aorta, the celiac axis, the superior mesenteric artery and/or the inferior mesenteric artery, sometimes with poor contrast opacification of the mesenteric vascular arcade, which is indicative of hypoperfusion and is better seen on maximum intensity projection (MIP) (Figure 6D). Accordingly, the segment of the injured small bowel reveals absent or decreased contrast enhancement and wall thickening, with target appearance due to mural edema. In ICU patients, most cases of acute mesenteric ischemia involve the large bowel alone (56%) and less frequently, both the large and small bowel (24%) [116]. The early phase of bowel ischemia may only show contracted gasless bowel that, when progressed, may transform into dilated intestinal loops with a paper-thin wall and air-fluid levels (Figure 6C). In the late phase, transmural infarction may lead to intestinal wall pneumatosis (Figure 6B), mesenteric venous gas, pneumoperitoneum due to perforation (Figure 6E), and free fluid in the abdominal cavity [111,112,117].

Imaging findings suggestive of viral colitis or terminal ileitis include circumferential bowel wall thickening with edema, mucosal hyperenhancement, mesenteric hypervascularity, fluid-filled mildly distended intestinal lumen and pericolic fat stranding, and in absence of filling defects is suggestive of thrombi in the abdominal arteries [44].

Pancreatic damage has been observed in 1–2% of mild cases and 17% of severe COVID-19 cases, including patients in ICU [118]. It has been speculated that pancreatic injury could be a consequence of both viral cytotoxicity on pancreatic islet cells and the severe immune response triggered by the infection [119]. Despite this, in most of these reports, the diagnosis was made only based on the elevation of pancreatic enzymes levels, which can be secondary to a multitude of causes, and radiological findings were missing. Cases of acute pancreatitis related to COVID-19 without additional pathology in the etiology have been described [120,121], including one case of necrotizing pancreatitis [122].

At present, COVID-19 related pancreatitis is a diagnosis of exclusion and requires the presence of at least two of the three revised Atlanta criteria (upper abdominal pain, serum lipase or amylase level >3× for the upper limit of normal and/or characteristic imaging features) [123].

In COVID-19 related pancreatitis, the pancreas appears diffusely enlarged on the CT scan, with ill-defined borders and a heterogeneous enhancement of the parenchyma. Stranding of the surrounding retroperitoneal fat is usually present and a streak of fluid along the anterior conal fascia and in the retro-mesenteric plane may be seen [111] (Figure 6A).

## 8. Renal Complications

Patients with COVID-19 could experience high rates of acute kidney injury (AKI), especially those in ICU, with rates of approximately 20–40% [124,125,126].

Since ACE-2 receptors are highly expressed in kidney tubules, renal injury is most likely caused by a direct virus-induced cytopathic effect, with acute tubular necrosis, interstitial inflammation and protein leakage in the Bowman’s capsule [127,128]; however, a systemic cytokine storm developing in severely ill patients may also play a role [129]. Moreover, there have been several reports describing renal infarcts due to the severe hypercoagulability state which can occur in COVID-19 patients [130].

AKI during COVID-19 infection is considered a negative prognostic factor for survival and patients may require renal replacement therapy [131].

**Figure 7:**
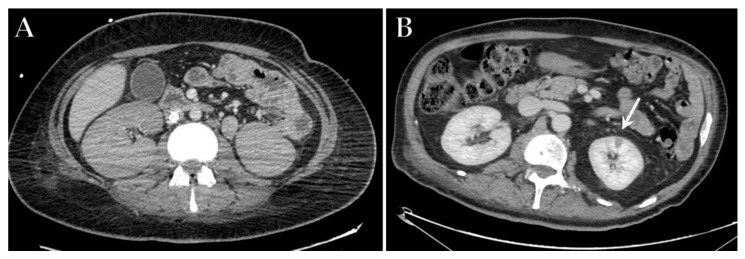
Axial contrast-enhanced CT image of an ICU patient with COVID-19 ARDS in ECMO treatment that developed kidney injury, showing enlarged kidney with a loss of corticomedullary differentiation (**A**). Axial contrast-enhanced CT image of a different patient with severe COVID-19 admitted to ICU demonstrating a wedge-shaped region of hypoattenuation in the left kidney (white arrow) consistent with renal infarction (**B**).

Abdominal CT with acquisition during the arterial and venous phases can easily detect AKI, showing the enlarged kidney with a loss of corticomedullary differentiation (Figure 7A). Renal infarctions appear as solitary or multiple, triangular-shaped areas of parenchyma with decreased perfusion and/or enhancement, with the apex pointing towards the medulla and base parallel to the subcapsular region [111,130,132] (Figure 7B). When a filling defect in the aorta or the renal arteries is not visible, microthrombi may be suspected, as they have been documented on histopathological samples [133].

Since impaired renal function increases patients’ susceptibility to contrast-induced nephropathy, contrast-enhanced imaging studies should be employed with caution and US, and despite its lower sensitivity, could represent a valid alternative.

## 9. Neurological Complications

Acute cerebrovascular events, such as acute ischemic stroke and intracranial hemorrhage (intraparenchymal and subarachnoid) are the most common neurological complications of COVID-19, but meningoencephalitis, encephalopathy and encephalomyelitis have also been reported [134].

COVID-19 neurological complications appear to involve both ischemic and hemorrhagic coagulation disturbances secondary to direct viral invasion through olfactory pathways or the bloodstream, with consequent endothelial damage [135]. Moreover, the exacerbated inflammatory response with the hypersecretion of cytokines, together with prolonged hypoxemia and a form of small vessel vasculitis, further increases the risk of acute cerebrovascular complications [136,137]. This could explain why cerebrovascular complications seem to affect patients with COVID-19 that are younger and have no history of vascular abnormality compared to patients with ischemic stroke/intracranial hemorrhages due to other causes [134].

Neurologic symptoms have been reported to be more common in patients admitted to ICU (84%), and are associated with a worse prognosis compared to patients with normal neurological examination [138]. Acute neurologic findings were recorded in 14% of patients on admission to the ICU [139], and stroke was demonstrated to be more frequent in this scenario compared to hospitalized patients (5–6% vs. 1–3%) [139,140]. In addition, despite the mortality benefit of ECMO demonstrated in patients with ARDS, the systemic anticoagulation required to reduce circuit clotting further increases the risk of neurologic complications, especially extensive intracerebral hemorrhages [141,142,143].

**Figure 8:**
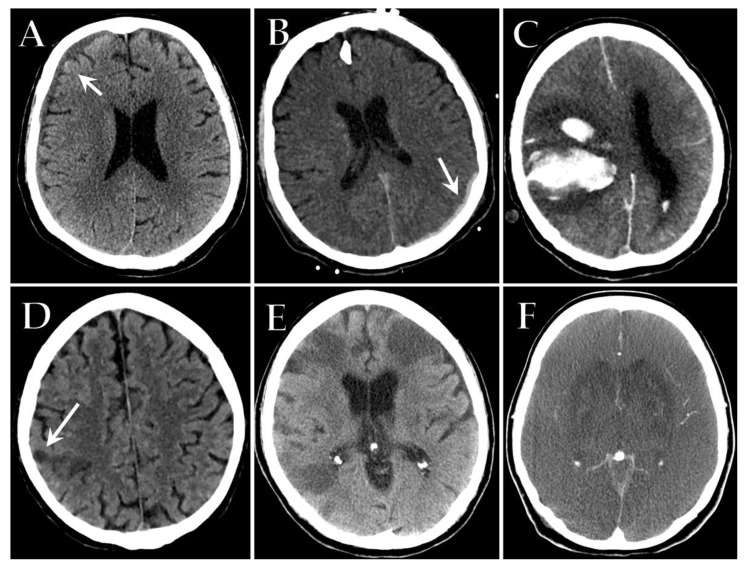
Axial unenhanced CT images of different patients with severe COVID-19 admitted to ICU showing spontaneous subacute right frontoparietal subdural hematoma (white arrow) (**A**), acute left parieto-occipital subdural hematoma (white arrow) (**B**) and massive right intracerebral hemorrhage with surrounding edema and significant midline shift (**C**). Axial unenhanced CT images show an acute ischemic stroke in the right parietal lobe (white arrow) (**D**) and multiple bilateral acute ischemic strokes (**E**) in two different COVID-19 patients admitted to ICU. Axial contrast-enhanced CT of a COVID-19 comatose patient reveals diffuse sulcal effacement with a decreased differentiation between gray and white matter, compatible with severe anoxic brain injury (**F**).

Imaging features of acute ischemic stroke in COVID-19 patients are similar to those described in non-COVID-19 patients, including abnormal hypoattenuation of the brain parenchyma, a loss of gray-white differentiation, and sulcal effacement on unenhanced CT (Figure 8D–F). After contrast media administration, an extensive vascular occlusive disease may be revealed, typically involving the large vessel [43].

Intracerebral hemorrhages may occur spontaneously in critically ill patients, particularly in the context of circulation instability, or as a hemorrhagic transformation of acute ischemic stroke. Hemorrhages in COVID-19 patients resemble those secondary to anticoagulant therapy, appearing as a markedly hyperdense area with surrounding edema at unenhanced CT. In severely ill ICU patients, hemorrhages may be massive, with extensive hemispheric involvement and/or with multiple hematomas occurring in both supra and infra-tentorial locations, sometimes with intraventricular extension [140,144] (Figure 8A–C).

Finally, recent studies have analyzed brain MRIs of critically ill patients with COVID-19 after their discharge and described diffuse leuko-encephalopathy and microhemorrhages as late complications of the prolonged hypoxemia and/or the hyperinflammatory state in about 25% of cases [145,146]. These reports suggest that COVID-19 may produce protracted neurological sequelae, but further studies are needed to confirm these results.

## 10. Sarcopenia

It has been reported that ICU patients with COVID-19 show significant reductions in skeletal muscle mass and strength during their hospitalization [147,148], with increased morbidity and mortality and persistence in about one-third of cases, even post-discharge [149].

The acute inflammatory state and the procoagulant state described in the severe form of COVID-19 may play a role in the onset of sarcopenia, causing an increased glucocorticoid and catecholamine production with consequent hypercatabolism and anabolic resistance [150,151]. This process perturbs muscle homeostasis and decreases muscle quality and quantity, especially at the level of respiratory muscles, impairing the ability to produce appropriate tidal volumes and to perform high force expulsive airway clearance maneuvers [152].

CT is considered the gold standard for investigating quantitative and qualitative changes in muscle and fat, especially in the trunk area. In fact, besides the mere quantification of the muscle mass, CT can evaluate the quality of muscle based on identifying the fat portion within the muscle through the evaluation of specific attenuation [153].

**Figure 9:**
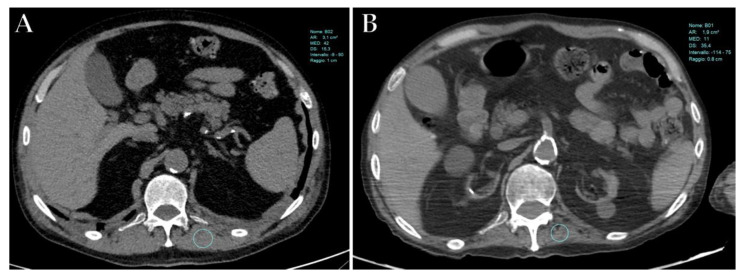
Axial unenhanced CT images of a patient with severe COVID-19 at the same level (T12), performed at the time of the ICU admission (**A**) and after a 3-month of ICU stay (**B**), demonstrates a reduction in muscle mass in the left paravertebral muscle (i.e., <30 HU) during hospitalization.

Giraudo et al. [154] have recently demonstrated that a value above 34 HU in the right paravertebral muscle at the level of T12 is a highly sensitive CT prognostic factor for ICU admission in COVID-19 patients (Figure 9A,B). Similarly, Schiaffino et al. [148] reported that paravertebral skeletal muscle mass at T12 and, especially, T5, can predict ICU admission and death. Finally, Damanti et al. [155] have demonstrated that muscle quality perturbations were predictive of the length of hospitalization and in-hospital mortality, as well as the need for reintubation after the discontinuation of mechanical ventilation.

## 11. Current Limits of Imaging in Critically Ill COVID-19 Patients

X-rays, and especially CT, are highly reproducible and easy to perform imaging techniques, which provide a rapid and accurate estimation of disease progression. However, their excessive use during the pandemic has placed a substantial burden on radiology departments, which had to adapt their workflow and organize workforces accordingly. Moreover, diagnostic imaging examinations have been complicated by the need for strict infection control and prevention practices to contain the risk of transmission to other patients or healthcare workers [156], which have caused both delays in patient management and the consumption of personal protection equipment [157]. Therefore, in a global pandemic setting, it is particularly important that imaging examinations are performed with specific clinical indications and only after careful consideration, primarily reserving them for detecting and/or monitoring the complications in hospitalized and severely ill COVID-19 patients.

Despite imaging being an integral part of healthcare, the spread of the pandemic to developing countries, including those in sub-Saharan Africa, has highlighted and further stressed the huge inequality in healthcare access around the world [158]. In fact, as the establishment of CT facilities is costly, it has been reported that developing countries have less than one CT scanner per one million inhabitants compared to almost forty scanners per one million inhabitants in high-income countries [159]. This shortage of equipment is also accompanied by a concomitant lack of trained healthcare manpower and reflects the higher risk of mortality in developing countries [160]. Therefore, while efforts should be made to expand the availability of critical imaging services to regions of great need, the providing of up-to-date evidence-based knowledge for diagnostic imaging may empower clinicians and radiologists in poor and underserved environments to choose proper imaging techniques with the aim to maximize patient benefits and ensure the optimal use of available financial resources.

## 12. Conclusions

During the COVID-19 pandemic, the growing request for prolonged pulmonary support for critically ill patients in ICU has increased the volume of imaging. Radiologists should be aware of the imaging features of COVID-19 ARDS and the complications of mechanical ventilation or other invasive supports. Moreover, it has been demonstrated that COVID-19 ARDS’ evolution may be heterogeneous and unpredictable, and may also potentially involve other organs and systems. Therefore, radiologists need to be familiar with these extra-pulmonary complications and, through reliable and constant monitoring, guide therapeutic decisions. Moreover, as more research is pursued and the pathophysiology of COVID-19 is increasingly understood, the role of imaging must evolve accordingly, expanding from the diagnosis and subsequent management of patients to prognosis prediction.

## Data Availability

Not applicable.

## References

[1] Simpson S., Kay F.U., Abbara S., Bhalla S., Chung J.H., Chung M., Henry T.S., Kanne J.P., Kligerman S., Ko J.P. (2020). Radiological Society of North America Expert Consensus Statement on Reporting Chest CT Findings Related to COVID-19. Endorsed by the Society of Thoracic Radiology, the American College of Radiology, and RSNA—Secondary Publication. J. Thorac. Imaging.

[2] Balacchi C., Brandi N., Ciccarese F., Coppola F., Lucidi V., Bartalena L., Parmeggiani A., Paccapelo A., Golfieri R. (2021). Comparing the first and the second waves of COVID-19 in Italy: Differences in epidemiological features and CT findings using a semi-quantitative score. Emerg. Radiol..

[3] Ciccarese F., Coppola F., Spinelli D., Galletta G.L., Lucidi V., Paccapelo A., DE Benedittis C., Balacchi C., Golfieri R. (2020). Diagnostic Accuracy of North America Expert Consensus Statement on Reporting CT Findings in Patients Suspected of Having COVID-19 Infection: An Italian Single-Center Experience. Radiol. Cardiothorac. Imaging.

[4] Angeli E., Dalto S., Marchese S., Setti L., Bonacina M., Galli F., Rulli E., Torri V., Monti C., Meroni R. (2021). Prognostic value of CT integrated with clinical and laboratory data during the first peak of the COVID-19 pandemic in Northern Italy: A nomogram to predict unfavorable outcome. Eur. J. Radiol..

[5] Tabatabaei S.M.H., Talari H., Moghaddas F., Rajebi H. (2020). CT Features and Short-term Prognosis of COVID-19 Pneumonia: A Single-Center Study from Kashan, Iran. Radiol. Cardiothorac. Imaging.

[6] Lo Bianco G., Di Pietro S., Mazzuca E., Imburgia A., Tarantino L., Accurso G., Benenati V., Vernuccio F., Bucolo C., Salomone S. (2020). Multidisciplinary Approach to the Diagnosis and In-Hospital Management of COVID-19 Infection: A Narrative Review. Front. Pharmacol..

[7] Pezoulas V.C., Kourou K.D., Papaloukas C., Triantafyllia V., Lampropoulou V., Siouti E., Papadaki M., Salagianni M., Koukaki E., Rovina N. (2021). A Multimodal Approach for the Risk Prediction of Intensive Care and Mortality in Patients with COVID-19. Diagnostics.

[8] Yang X., Yu Y., Xu J., Shu H., Xia J., Liu H., Wu Y., Zhang L., Yu Z., Fang M. (2020). Clinical course and outcomes of critically ill patients with SARS-CoV-2 pneumonia in Wuhan, China: A single-centered, retrospective, observational study. Lancet Respir. Med..

[9] Bhimraj A., Morgan R.L., Shumaker A.H., Lavergne V., Baden L., Cheng V.C.-C., Edwards K.M., Gandhi R., Muller W.J., O’Horo J.C. (2020). Infectious Diseases Society of America Guidelines on the Treatment and Management of Patients with (COVID-19). Clin. Infect. Dis..

[10] Bai C., Chotirmall S.H., Rello J., Alba G.A., Ginns L.C., Krishnan J.A., Rogers R., Bendstrup E., Burgel P.-R., Chalmers J.D. (2020). Updated guidance on the management of COVID-19: From an American Thoracic Society/European Respiratory Society coordinated International Task Force (29 July 2020). Eur. Respir. Rev..

[11] Wang I.E., Cooper G., Mousa S.A. (2021). Diagnostic Approaches for COVID-19 and Its Associated Complications. Diagnostics.

[12] Cau R., Falaschi Z., Paschè A., Danna P., Arioli R., Arru C.D., Zagaria D., Tricca S., Suri J.S., Karla M.K. (2021). Computed tomography findings of COVID-19 pneumonia in Intensive Care Unit-patients. J. Public Health Res..

[13] Franco C., Facciolongo N., Tonelli R., Dongilli R., Vianello A., Pisani L., Scala R., Malerba M., Carlucci A., Negri E.A. (2020). Feasibility and clinical impact of out-of-ICU noninvasive respiratory support in patients with COVID-19-related pneumonia. Eur. Respir. J..

[14] Wilcox S.R., Condella A. (2021). Emergency Department Management of Severe Hypoxemic Respiratory Failure in Adults with COVID-19. J. Emerg. Med..

[15] Cornejo R.A., Díaz J.C., Tobar E., Bruhn A., Ramos C.A., González R.A., Repetto C.A., Romero C.M., Gálvez L.R., Llanos O. (2013). Effects of Prone Positioning on Lung Protection in Patients with Acute Respiratory Distress Syndrome. Am. J. Respir. Crit. Care Med..

[16] Gattinoni L., Busana M., Giosa L., Macrì M.M., Quintel M. (2019). Prone Positioning in Acute Respiratory Distress Syndrome. Semin. Respir. Crit. Care Med..

[17] Scaramuzzo G., Gamberini L., Tonetti T., Zani G., Ottaviani I., Mazzoli C.A., Capozzi C., Giampalma E., Reggiani M.L.B., Bertellini E. (2021). Sustained oxygenation improvement after first prone positioning is associated with liberation from mechanical ventilation and mortality in critically ill COVID-19 patients: A cohort study. Ann. Intensiv. Care.

[18] Pan C., Chen L., Lu C., Zhang W., Xia J.-A., Sklar M.C., Du B., Brochard L., Qiu H. (2020). Lung Recruitability in COVID-19–associated Acute Respiratory Distress Syndrome: A Single-Center Observational Study. Am. J. Respir. Crit. Care Med..

[19] Gattinoni L., Chiumello D., Caironi P., Busana M., Romitti F., Brazzi L., Camporota L. (2020). COVID-19 pneumonia: Different respiratory treatments for different phenotypes?. Intensiv. Care Med..

[20] Bonnesen B., Jensen J.-U.S., Jeschke K.N., Mathioudakis A.G., Corlateanu A., Hansen E.F., Weinreich U.M., Hilberg O., Sivapalan P. (2021). Management of COVID-19-Associated Acute Respiratory Failure with Alternatives to Invasive Mechanical Ventilation: High-Flow Oxygen, Continuous Positive Airway Pressure, and Noninvasive Ventilation. Diagnostics.

[21] Gresser E., Rueckel J., Puhr-Westerheide D., Schwarze V., Fink N., Kunz W.G., Wassilowsky D., Irlbeck M., Ricke J., Ingrisch M. (2020). Prognostic Value of Admission Chest CT Findings for Invasive Ventilation Therapy in COVID-19 Pneumonia. Diagnostics.

[22] Rola P., Farkas J., Spiegel R., Kyle-Sidell C., Weingart S., Duggan L., Garrone M., Thomas A. (2020). Rethinking the early intubation paradigm of COVID-19: Time to change gears?. Clin. Exp. Emerg. Med..

[23] McGuinness G., Zhan C., Rosenberg N., Azour L., Wickstrom M., Mason D.M., Thomas K.M., Moore W.H. (2020). Increased Incidence of Barotrauma in Patients with COVID-19 on Invasive Mechanical Ventilation. Radiology.

[24] Grasselli G., Zangrillo A., Zanella A., Antonelli M., Cabrini L., Castelli A., Cereda D., Coluccello A., Foti G., Fumagalli R. (2020). Baseline Characteristics and Outcomes of 1591 Patients Infected With SARS-CoV-2 Admitted to ICUs of the Lombardy Region, Italy. JAMA.

[25] Ñamendys-Silva S.A. (2020). ECMO for ARDS due to COVID-19. Heart Lung.

[26] Ma X., Liang M., Ding M., Liu W., Ma H., Zhou X., Ren H. (2020). Extracorporeal Membrane Oxygenation (ECMO) in Critically Ill Patients with Coronavirus Disease 2019 (COVID-19) Pneumonia and Acute Respiratory Distress Syndrome (ARDS). Med. Sci. Monit..

[27] Douraghi-Zadeh D., Logaraj A., Lazoura O., Downey K., Gill S., Finney S.J., Padley S. (2021). Extracorporeal membrane oxygenation (ECMO): Radiographic appearances, complications and imaging artefacts for radiologists. J. Med. Imaging Radiat. Oncol..

[28] Gabelloni M., Faggioni L., Cioni D., Mendola V., Falaschi Z., Coppola S., Corradi F., Isirdi A., Brandi N., Coppola F. (2022). Extracorporeal membrane oxygenation (ECMO) in COVID-19 patients: A pocket guide for radiologists. Radiol. Med..

[29] Zheng M.-H., Feng G., Liu W., Targher G., Byrne C.D., Zheng M. (2021). Extrapulmonary complications of COVID-19: A multisystem disease?. J. Med. Virol..

[30] Belletti A., Todaro G., Valsecchi G., Losiggio R., Palumbo D., Landoni G., Zangrillo A. (2022). Barotrauma in Coronavirus Disease 2019 Patients Undergoing Invasive Mechanical Ventilation: A Systematic Literature Review. Crit. Care Med..

[31] Papi C., Spagni G., Alexandre A., Calabresi P., Della Marca G., Broccolini A. (2020). Unprotected stroke management in an undiagnosed case of Severe Acute Respiratory Syndrome Coronavirus 2 infection. J. Stroke Cerebrovasc. Dis..

[32] Ranieri V.M., Rubenfeld G.D., Thompson B.T., Ferguson N.D., Caldwell E., Fan E., Camporota L., Slutsky A.S., ARDS Definition of Task Force (2012). Acute Respiratory Distress Syndrome: The Berlin Definition. JAMA.

[33] Arrivé F., Coudroy R., Thille A.W. (2021). Early Identification and Diagnostic Approach in Acute Respiratory Distress Syndrome (ARDS). Diagnostics.

[34] Wong K.T., Antonio G.E., Hui D., Lee N., Yuen E.H.Y., Wu A., Leung C.B., Rainer T., Cameron P., Chung S.S.C. (2003). Severe Acute Respiratory Syndrome: Radiographic Appearances and Pattern of Progression in 138 Patients. Radiology.

[35] Valente T., Lassandro F., Marino M., Squillante F., Aliperta M., Muto R. (2012). H1N1 pneumonia: Our experience in 50 patients with a severe clinical course of novel swine-origin influenza A (H1N1) virus (S-OIV). Radiol. Med..

[36] Franquet T., Jeong Y.J., Lam H.Y.S., Wong H.Y.F., Chang Y.-C., Chung M.J., Lee K.S. (2020). Imaging findings in coronavirus infections: SARS-CoV, MERS-CoV, and SARS-CoV-2. Br. J. Radiol..

[37] Sheard S., Rao P., Devaraj A. (2012). Imaging of Acute Respiratory Distress Syndrome. Respir. Care.

[38] Gosangi B., Rubinowitz A.N., Irugu D., Gange C., Bader A., Cortopassi I. (2022). COVID-19 ARDS: A review of imaging features and overview of mechanical ventilation and its complications. Emerg. Radiol..

[39] Gibson P.G., Qin L., Puah S.H. (2020). COVID-19 acute respiratory distress syndrome (ARDS): Clinical features and differences from typical pre-COVID-19 ARDS. Med. J. Aust..

[40] Gattinoni L., Coppola S., Cressoni M., Busana M., Rossi S., Chiumello D. (2020). COVID-19 Does Not Lead to a “Typical” Acute Respiratory Distress Syndrome. Am. J. Respir. Crit. Care Med..

[41] Li X., Ma X. (2020). Acute respiratory failure in COVID-19: Is it “typical” ARDS?. Crit. Care.

[42] Helms J., Tacquard C., Severac F., Leonard-Lorant I., Ohana M., Delabranche X., Merdji H., Clere-Jehl R., Schenck M., Gandet F.F. (2020). High risk of thrombosis in patients with severe SARS-CoV-2 infection: A multicenter prospective cohort study. Intensive Care Med..

[43] Goldberg M.F., Goldberg M.F. (2020). Neuroradiologic manifestations of COVID-19: What the emergency radiologist needs to know. Emerg. Radiol..

[44] Bhayana R., Som A., Li M.D., Carey D.E., Anderson M.A., Blake M.A., Catalano O., Gee M.S., Hahn P.F., Harisinghani M. (2020). Abdominal Imaging Findings in COVID-19: Preliminary Observations. Radiology.

[45] Gamberini L., Tonetti T., Spadaro S., Zani G., Mazzoli C.A., Capozzi C., Giampalma E., Reggiani M.L.B., Bertellini E., Castelli A. (2020). Factors influencing liberation from mechanical ventilation in coronavirus disease 2019: Multicenter observational study in fifteen Italian ICUs. J. Intensiv. Care.

[46] Wu C., Chen X., Cai Y., Xia J., Zhou X., Xu S., Huang H., Zhang L., Zhou X., Du C. (2020). Risk Factors Associated With Acute Respiratory Distress Syndrome and Death in Patients With Coronavirus Disease 2019 Pneumonia in Wuhan, China. JAMA Intern. Med..

[47] Zhang P., Li J., Liu H., Han N., Ju J., Kou Y., Chen L., Jiang M., Pan F., Zheng Y. (2020). Long-term bone and lung consequences associated with hospital-acquired severe acute respiratory syndrome: A 15-year follow-up from a prospective cohort study. Bone Res..

[48] Das K.M., Lee E.Y., Singh R., Enani M.A., Al Dossari K., Van Gorkom K., Larsson S.G., Langer R.D. (2017). Follow-up chest radiographic findings in patients with MERS-CoV after recovery. Indian J. Radiol. Imaging.

[49] Mineo G., Ciccarese F., Modolon C., Landini M.P., Valentino M., Zompatori M. (2011). Post-ARDS pulmonary fibrosis in patients with H1N1 pneumonia: Role of follow-up CT. La Radiol. Med..

[50] Urciuoli L., Guerriero E. (2020). Chest CT Findings after 4 Months from the Onset of COVID-19 Pneumonia: A Case Series. Diagnostics.

[51] González J., Benítez I.D., Carmona P., Santisteve S., Monge A., Moncusí-Moix A., Gort-Paniello C., Pinilla L., Carratalá A., Zuil M. (2021). Pulmonary Function and Radiologic Features in Survivors of Critical COVID-19: A 3-Month Prospective Cohort. Chest.

[52] Garfield J.M. (2021). One-year Multidisciplinary Follow-Up of COVID-19 Patients Requiring Invasive Mechanical Ventilation. J. Cardiothorac. Vasc. Anesthesia.

[53] Shrestha D.B., Sedhai Y.R., Budhathoki P., Adhikari A., Pokharel N., Dhakal R., Kafle S., Mir W.A.Y., Acharya R., Kashiouris M.G. (2022). Pulmonary barotrauma in COVID-19: A systematic review and meta-analysis. Ann. Med. Surg..

[54] Gupta V.K., Alkandari B.M., Mohammed W., Tobar A.M., Abdelmohsen M.A. (2020). Ventilator associated lung injury in severe COVID-19 pneumonia patients—Case Reports: Ventilator associated lung injury in COVID-19. Eur. J. Radiol. Open.

[55] Udwadia Z.F., Toraskar K.K., Pinto L., Mullerpatan J., Wagh H.D., Mascarenhas J.M., Gandhi B.M., Tripathi A., Sunavala A., Agrawal U. (2021). Increased frequency of pneumothorax and pneumomediastinum in COVID-19 patients admitted in the ICU: A multicentre study from Mumbai, India. Clin. Med..

[56] Chopra A., Al-Tarbsheh A.H., Shah N.J., Yaqoob H., Hu K., Feustel P.J., Ortiz-Pacheco R., Patel K.M., Oweis J., Kozlova N. (2021). Pneumothorax in critically ill patients with COVID-19 infection: Incidence, clinical characteristics and outcomes in a case control multicenter study. Respir. Med..

[57] Protti A., Greco M., Filippini M., Vilardo A.M., Langer T., Villa M., Frutos-Vivar F., Santini A., Caruso P.F., Spano S. (2021). Barotrauma in mechanically ventilated patients with Coronavirus disease 2019: A survey of 38 hospitals in Lombardy, Italy. Minerva Anestesiol..

[58] Wang X.H., Duan J., Han X., Liu X., Zhou J., Wang X., Zhu L., Mou H., Guo S. (2021). High incidence and mortality of pneumothorax in critically Ill patients with COVID-19. Heart Lung.

[59] Alhumaid S., Al Mutair A., Alghazal H.A., Alhaddad A.J., Al-Helal H., Al Salman S.A., Alali J., Almahmoud S., Alhejy Z.M., Albagshi A.A. (2021). Extracorporeal membrane oxygenation support for SARS-CoV-2: A multi-centered, prospective, observational study in critically ill 92 patients in Saudi Arabia. Eur. J. Med. Res..

[60] Martinelli A.W., Ingle T., Newman J., Nadeem I., Jackson K., Lane N.D., Melhorn J., Davies H.E., Rostron A.J., Adeni A. (2020). COVID-19 and pneumothorax: A multicentre retrospective case series. Eur. Respir. J..

[61] Zantah M., Castillo E.D., Townsend R., Dikengil F., Criner G.J. (2020). Pneumothorax in COVID-19 disease- incidence and clinical characteristics. Respir. Res..

[62] Kohli A., Hande P.C., Chugh S. (2021). Role of chest radiography in the management of COVID-19 pneumonia: An overview and correlation with pathophysiologic changes. Indian J. Radiol. Imaging.

[63] Ziter F.M., Westcott J.L. (1981). Supine subpulmonary pneumothorax. Am. J. Roentgenol..

[64] Akdogan R.E., Mohammed T., Syeda A., Jiwa N., Ibrahim O., Mutneja R. (2021). Pneumothorax in Mechanically Ventilated Patients with COVID-19 Infection. Case Rep. Crit. Care.

[65] Palumbo D., Zangrillo A., Belletti A., Guazzarotti G., Calvi M.R., Guzzo F., Pennella R., Monti G., Gritti C., Marmiere M. (2021). A radiological predictor for pneumomediastinum/pneumothorax in COVID-19 ARDS patients. J. Crit. Care.

[66] Bonato M., Fraccaro A., Landini N., Zanardi G., Catino C., Savoia F., Malacchini N., Zeraj F., Peditto P., Catalanotti V. (2021). Pneumothorax and/or Pneumomediastinum Worsens the Prognosis of COVID-19 Patients with Severe Acute Respiratory Failure: A Multicenter Retrospective Case-Control Study in the North-East of Italy. J. Clin. Med..

[67] Gahona C.C.T., Raj K., Bhandari K., Nuguru S., Bukhari A. (2020). Subcutaneous Emphysema in Patients With COVID-19 Infection: A Report of Three Cases. Cureus.

[68] Scacciavillani R., Iannaccone G., Del Buono M.G., Bello G. (2020). Pneumopericardium following mechanical ventilation in COVID-19 pneumonia. Eur. Heart J.-Case Rep..

[69] Belletti A., Palumbo D., Zangrillo A., Fominskiy E.V., Franchini S., Dell’Acqua A., Marinosci A., Monti G., Vitali G., Colombo S. (2021). Predictors of Pneumothorax/Pneumomediastinum in Mechanically Ventilated COVID-19 Patients. J. Cardiothorac. Vasc. Anesth..

[70] Lemmers D.H., Abu Hilal M., Bnà C., Prezioso C., Cavallo E., Nencini N., Crisci S., Fusina F., Natalini G. (2020). Pneumomediastinum and subcutaneous emphysema in COVID-19: Barotrauma or lung frailty?. ERJ Open Res..

[71] Bejvan S.M., Godwin J.D. (1996). Pneumomediastinum: Old signs and new signs. AJR Am. J. Roentgenol..

[72] Zylak C.M., Standen J.R., Barnes G.R., Zylak C.J. (2000). Pneumomediastinum Revisited. RadioGraphics.

[73] Kouritas V.K., Papagiannopoulos K., Lazaridis G., Baka S., Mpoukovinas I., Karavasilis V., Lampaki S., Kioumis I., Pitsiou G., Papaiwannou A. (2015). Pneumomediastinum. J. Thorac. Dis..

[74] Rouyer M., Strazzulla A., Youbong T., Tarteret P., Pitsch A., de Pontfarcy A., Cassard B., Vignier N., Pourcine F., Jochmans S. (2021). Ventilator-Associated Pneumonia in COVID-19 Patients: A Retrospective Cohort Study. Antibiotics.

[75] Wicky P.-H., Niedermann M.S., Timsit J.-F. (2021). Ventilator-associated pneumonia in the era of COVID-19 pandemic: How common and what is the impact?. Crit. Care.

[76] Maes M., Higginson E., Pereira-Dias J., Curran M.D., Parmar S., Khokhar F., Cuchet-Lourenço D., Lux J., Sharma-Hajela S., Ravenhill B. (2021). Ventilator-associated pneumonia in critically ill patients with COVID-19. Crit. Care.

[77] Lansbury L., Lim B., Baskaran V., Lim W.S. (2020). Co-infections in people with COVID-19: A systematic review and meta-analysis. J. Infect..

[78] Razazi K., Arrestier R., Haudebourg A.F., Benelli B., Carteaux G., Decousser J.-W., Fourati S., Woerther P.L., Schlemmer F., Charles-Nelson A. (2020). Risks of ventilator-associated pneumonia and invasive pulmonary aspergillosis in patients with viral acute respiratory distress syndrome related or not to Coronavirus 19 disease. Crit. Care.

[79] Blonz G., Kouatchet A., Chudeau N., Pontis E., Lorber J., Lemeur A., Planche L., Lascarrou J.-B., Colin G. (2021). Epidemiology and microbiology of ventilator-associated pneumonia in COVID-19 patients: A multicenter retrospective study in 188 patients in an un-inundated French region. Crit. Care.

[80] Moretti M., Van Laethem J., Minini A., Pierard D., Malbrain M.L. (2021). Ventilator-associated bacterial pneumonia in coronavirus 2019 disease, a retrospective monocentric cohort study. J. Infect. Chemother..

[81] Póvoa H.C.C., Chianca G.C., Iorio N.L.P.P. (2020). COVID-19: An Alert to Ventilator-Associated Bacterial Pneumonia. Infect. Dis. Ther..

[82] Marchiori E., Nobre L.F., Hochhegger B., Zanetti G. (2021). Pulmonary cavitation in patients with COVID-19. Clin. Imaging.

[83] Bartoletti M., Pascale R., Cricca M., Rinaldi M., Maccaro A., Bussini L., Fornaro G., Tonetti T., Pizzilli G., Francalanci E. (2020). Epidemiology of Invasive Pulmonary Aspergillosis Among Intubated Patients With COVID-19: A Prospective Study. Clin. Infect. Dis..

[84] Koehler P., Cornely O.A., Böttiger B.W., Dusse F., Eichenauer D.A., Fuchs F., Hallek M., Jung N., Klein F., Persigehl T. (2020). COVID-19 associated pulmonary aspergillosis. Mycoses.

[85] Song G., Liang G., Liu W. (2020). Fungal Co-infections Associated with Global COVID-19 Pandemic: A Clinical and Diagnostic Perspective from China. Mycopathologia.

[86] Patti R.K., Dalsania N.R., Somal N., Sinha A., Mehta S., Ghitan M., Seneviratne C., Kupfer Y. (2020). Subacute Aspergillosis “Fungal Balls” Complicating COVID-19. J. Investig. Med. High Impact Case Rep..

[87] Blot S.I., Taccone F.S., Van den Abeele A.-M., Bulpa P., Meersseman W., Brusselaers N., Dimopoulos G., Paiva J.A., Misset B., Rello J. (2012). A Clinical Algorithm to Diagnose Invasive Pulmonary Aspergillosis in Critically Ill Patients. Am. J. Respir. Crit. Care Med..

[88] Jenks J.D., Nam H.H., Hoenigl M. (2021). Invasive aspergillosis in critically ill patients: Review of definitions and diagnostic approaches. Mycoses.

[89] Aggarwal A., Tandon A., Bhatt S., Aggarwal A., Dagar S., Bansal H. (2021). COVID19 pneumonia with cavitation and cystic lung changes: Multi-detector computed tomography spectrum of a gamut of etiologies. BJR Open.

[90] Zoumot Z., Bonilla M.-F., Wahla A.S., Shafiq I., Uzbeck M., El-Lababidi R.M., Hamed F., Abuzakouk M., ElKaissi M. (2021). Pulmonary cavitation: An under-recognized late complication of severe COVID-19 lung disease. BMC Pulm. Med..

[91] Brandi N., Bartalena L., Mosconi C., Golfieri R. (2021). A unique case of miliary pulmonary tuberculosis induced by bacillus Calmette-Guérin intravesical instillation with COVID-19 superinfection. S. Afr. J. Radiol..

[92] Amaral L.T.W., Beraldo G.L., Brito V.M., Rosa M.E.E., De Matos M.J.R., Fonseca E.K.U.N., Yokoo P., Silva M.M.A., Teles G.B.D.S., Shoji H. (2020). Lung cavitation in COVID-19: Co-infection complication or rare evolution?. Einstein.

[93] Adams H.J., Kwee T.C., Yakar D., Hope M.D., Kwee R.M. (2020). Chest CT Imaging Signature of Coronavirus Disease 2019 Infection: In Pursuit of the Scientific Evidence. Chest.

[94] Moll M., Zon R.L., Sylvester K.W., Chen E.C., Cheng V., Connell N., Fredenburgh L.E., Baron R.M., Cho M.H., Woolley A.E. (2020). VTE in ICU Patients With COVID-19. Chest.

[95] Llitjos J.-F., Leclerc M., Chochois C., Monsallier J.-M., Ramakers M., Auvray M., Merouani K. (2020). High incidence of venous thromboembolic events in anticoagulated severe COVID-19 patients. J. Thromb. Haemost..

[96] Poissy J., Goutay J., Caplan M., Parmentier-Decrucq E., Duburcq T., Lassalle F., Jeanpierre E., Rauch A., Labreuche J., Susen S. (2020). Pulmonary Embolism in Patients With COVID-19: Awareness of an Increased Prevalence. Circulation.

[97] Mirsadraee S., Gorog D.A., Mahon C.F., Rawal B., Semple T.R., Nicol E.D., Arachchillage D.R.J., Devaraj A., Price S., Desai S.R. (2021). Prevalence of Thrombotic Complications in ICU-Treated Patients with Coronavirus Disease 2019 Detected with Systematic CT Scanning. Crit. Care Med..

[98] Suh Y.J., Hong H., Ohana M., Bompard F., Revel M.-P., Valle C., Gervaise A., Poissy J., Susen S., Hékimian G. (2021). Pulmonary Embolism and Deep Vein Thrombosis in COVID-19: A Systematic Review and Meta-Analysis. Radiology.

[99] Longhitano Y., Racca F., Zanza C., Piccioni A., Audo A., Muncinelli M., Santi R., Kozel D., Geraci C., Taverna M. (2020). Venous thromboembolism in critically ill patients affected by ARDS related to COVID-19 in Northern-West Italy. Eur. Rev. Med. Pharmacol. Sci..

[100] Lamarche Y., Chow B., Bédard A., Johal N., Kaan A., Humphries K.H., Cheung A. (2010). Thromboembolic events in patients on extracorporeal membrane oxygenation without anticoagulation. Innovations.

[101] Schmidt M., Hajage D., Lebreton G., Monsel A., Voiriot G., Levy D., Baron E., Beurton A., Chommeloux J., Meng P. (2020). Extracorporeal membrane oxygenation for severe acute respiratory distress syndrome associated with COVID-19: A retrospective cohort study. Lancet Respir. Med..

[102] Gidaro A., Vailati D., Gemma M., Lugli F., Casella F., Cogliati C., Canelli A., Cremonesi N., Monolo D., Cordio G. (2021). Retrospective survey from vascular access team Lombardy net in COVID-19 era. J. Vasc. Access.

[103] Lang M., Som A., Carey D., Reid N., Mendoza D.P., Flores E.J., Li M.D., Shepard J.-A.O., Little B.P. (2020). Pulmonary Vascular Manifestations of COVID-19 Pneumonia. Radiol. Cardiothorac. Imaging.

[104] Lee E.E., Gong A.J., Gawande R.S., Fishman E.K., Vadvala H.V. (2022). Vascular findings in CTA body and extremity of critically ill COVID-19 patients: Commonly encountered vascular complications with review of literature. Emerg. Radiol..

[105] Faqihi F., Alharthy A., Balhamar A., Nasim N., AlAnezi K., Alaklobi F., Memish Z.A., Blaivas M., Alqahtani S.A., Karakitsos D. (2021). A Retrospective Analysis of Thromboembolic Phenomena in Mechanically Ventilated Patients with COVID-19. Crit. Care Res. Pract..

[106] Palumbo D., Guazzarotti G., De Cobelli F. (2020). Spontaneous Major Hemorrhage in COVID-19 Patients: Another Brick in the Wall of SARS-CoV-2–Associated Coagulation Disorders?. J. Vasc. Interv. Radiol..

[107] Angileri S.A., Petrillo M., Di Meglio L., Arrichiello A., Rodà G.M., Ierardi A.M., Uberoi R., Carrafiello G. (2020). Adverse Events in Coronavirus Disease Patients Management: A Pictorial Essay. J. Clin. Imaging Sci..

[108] Bargellini I., Cervelli R., Lunardi A., Scandiffio R., Daviddi F., Giorgi L., Cicorelli A., Crocetti L., Cioni R. (2020). Spontaneous Bleedings in COVID-19 Patients: An Emerging Complication. Cardiovasc. Interv. Radiol..

[109] Abdelmohsen M.A., Alkandari B.M., Razek A.A.K.A., Tobar A.M., Gupta V.K., Elsebaie N. (2021). Abdominal Computed Tomography Angiography and Venography in Evaluation of Hemorrhagic and Thrombotic lesions in Hospitalized COVID-19 patients. Clin. Imaging.

[110] Revzin M.V., Raza S., Srivastava N.C., Warshawsky R., D’Agostino C., Malhotra A., Bader A.S., Patel R.D., Chen K., Kyriakakos C. (2020). Multisystem Imaging Manifestations of COVID-19, Part 2: From Cardiac Complications to Pediatric Manifestations. RadioGraphics.

[111] Vaidya T., Nanivadekar A., Patel R. (2021). Imaging spectrum of abdominal manifestations of COVID-19. World J. Radiol..

[112] Muñoz C.A., Zapata M., Gómez C.I., Pino L.F., Herrera M.A., González-Hadad A. (2021). Large intestinal perforation secondary to COVID-19: A case report. Int. J. Surg. Case Rep..

[113] Keshavarz P., Rafiee F., Kavandi H., Goudarzi S., Heidari F., Gholamrezanezhad A. (2020). Ischemic gastrointestinal complications of COVID-19: A systematic review on imaging presentation. Clin. Imaging.

[114] Kaafarani H.M.A., El Moheb M., Hwabejire J.O., Naar L., Christensen M.A., Breen K., Gaitanidis A., Alser O., Mashbari H., Bankhead-Kendall B. (2020). Gastrointestinal Complications in Critically Ill Patients with COVID-19. Ann. Surg..

[115] El Moheb M., Naar L., Christensen M.A., Kapoen C., Maurer L.R., Farhat M., Kaafarani H.M.A. (2020). Gastrointestinal Complications in Critically Ill Patients with and without COVID-19. JAMA.

[116] Serban D., Tribus L.C., Vancea G., Stoian A.P., Dascalu A.M., Suceveanu A.I., Tanasescu C., Costea A.C., Tudosie M.S., Tudor C. (2021). Acute Mesenteric Ischemia in COVID-19 Patients. J. Clin. Med..

[117] Caruso D., Zerunian M., Pucciarelli F., Lucertini E., Bracci B., Polidori T., Guido G., Polici M., Rucci C., Iannicelli E. (2021). Imaging of abdominal complications of COVID-19 infection. BJR Open.

[118] Bozdag A., Eroglu Y., Tartar A.S., Bozdag P.G., Aglamis S. (2021). Pancreatic Damage and Radiological Changes in Patients with COVID-19. Cureus.

[119] Wang F., Wang H., Fan J., Zhang Y., Wang H., Zhao Q. (2020). Pancreatic Injury Patterns in Patients With Coronavirus Disease 19 Pneumonia. Gastroenterology.

[120] Kurihara Y., Maruhashi T., Wada T., Osada M., Oi M., Yamaoka K., Asari Y. (2020). Pancreatitis in a Patient with Severe Coronavirus Disease Pneumonia Treated with Veno-venous Extracorporeal Membrane Oxygenation. Intern. Med..

[121] Hadi A., Werge M., Kristiansen K.T., Pedersen U.G., Karstensen J.G., Novovic S., Gluud L.L. (2020). Coronavirus Disease-19 (COVID-19) associated with severe acute pancreatitis: Case report on three family members. Pancreatology.

[122] Kumaran N.K., Karmakar B.K., Taylor O.M. (2020). Coronavirus disease-19 (COVID-19) associated with acute necrotising pancreatitis (ANP). BMJ Case Rep..

[123] Kataria S., Sharif A., Rehman A.U., Ahmed Z., Hanan A. (2020). COVID-19 Induced Acute Pancreatitis: A Case Report and Literature Review. Cureus.

[124] Ronco C., Reis T., Husain-Syed F. (2020). Management of acute kidney injury in patients with COVID-19. Lancet Respir. Med..

[125] Wang P., Tan X., Li Q., Qian M., Cheng A., Ma B., Zhang X., Guo C., Sheng M., Yi M. (2021). Extra-pulmonary complications of 45 critically ill patients with COVID-19 in Yichang, Hubei province, China: A single-centered, retrospective, observation study. Medicine.

[126] Yuan H., Liu J., Gao Z., Hu F. (2021). Clinical Features and Outcomes of Acute Kidney Injury in Patients Infected with COVID-19 in Xiangyang, China. Blood Purif..

[127] Batlle D., Soler M.J., Sparks M.A., Hiremath S., South A.M., Welling P.A., Swaminathan S. (2020). COVID-19 and ACE2 in Cardiovascular, Lung, and Kidney Working Group Acute Kidney Injury in COVID-19: Emerging Evidence of a Distinct Pathophysiology. J. Am. Soc. Nephrol..

[128] Liu Q., Song N.C., Zheng Z.K., Li J.S., Li S.K. (2020). Laboratory findings and a combined multifactorial approach to predict death in critically ill patients with COVID-19: A retrospective study. Epidemiol. Infect..

[129] Ahmad Z., Goswami S., Paneerselvam A., Kabilan K., Choudhury H., Roy A., Guleria R., Soni K.D., Baruah U., Das C.J. (2021). Imaging of Coronavirus Disease 2019 Infection From Head to Toe: A Primer for the Radiologist. Curr. Probl. Diagn. Radiol..

[130] Akin I.B., Altay C., Kutsoylu O.E., Secil M. (2021). Possible radiologic renal signs of COVID-19. Abdom. Radiol..

[131] Ronco C., Reis T. (2020). Kidney involvement in COVID-19 and rationale for extracorporeal therapies. Nat. Rev. Nephrol..

[132] Post A., den Deurwaarder E.S.G., Bakker S.J.L., de Haas R.J., van Meurs M., Gansevoort R.T., Berger S.P. (2020). Kidney Infarction in Patients With COVID-19. Am. J. Kidney Dis..

[133] Su H., Yang M., Wan C., Yi L.-X., Tang F., Zhu H.-Y., Yi F., Yang H.-C., Fogo A.B., Nie X. (2020). Renal histopathological analysis of 26 postmortem findings of patients with COVID-19 in China. Kidney Int..

[134] Alves V.D.P.V., Altoé A., Veloso V., Ferreira C.L.S., Ventura N., Corrêa D.G. (2021). Computed tomography features of cerebrovascular complications in intensive care unit patients with severe COVID-19. Radiol. Bras..

[135] Sklinda K., Dorobek M., Wasilewski P.G., Dreżewski K., Dȩbicka M., Walecki J., Mruk B. (2021). Radiological Manifestation of Neurological Complications in the Course of SARS-CoV-2 Infection. Front. Neurol..

[136] Choi Y., Lee M.K. (2020). Neuroimaging findings of brain MRI and CT in patients with COVID-19: A systematic review and meta-analysis. Eur. J. Radiol..

[137] Li Y.C., Bai W.Z., Hashikawa T. (2020). The neuroinvasive potential of SARS-CoV2 may play a role in the respiratory failure of COVID-19 patients. J. Med. Virol..

[138] Helms J., Kremer S., Merdji H., Schenck M., Severac F., Clere-Jehl R., Studer A., Radosavljevic M., Kummerlen C., Monnier A. (2020). Delirium and encephalopathy in severe COVID-19: A cohort analysis of ICU patients. Crit. Care.

[139] Helms J., Kremer S., Merdji H., Clere-Jehl R., Schenck M., Kummerlen C., Collange O., Boulay C., Fafi-Kremer S., Ohana M. (2020). Neurologic Features in Severe SARS-CoV-2 Infection. N. Engl. J. Med..

[140] Vogrig A., Gigli G.L., Bnà C., Morassi M. (2020). Stroke in patients with COVID-19: Clinical and neuroimaging characteristics. Neurosci. Lett..

[141] Heman-Ackah S.M., Su Y.S., Spadola M., Petrov D., Chen H.I., Schuster J., Lucas T. (2020). Neurologically Devastating Intraparenchymal Hemorrhage in COVID-19 Patients on Extracorporeal Membrane Oxygenation: A Case Series. Neurosurgery.

[142] Peek G.J., Mugford M., Tiruvoipati R., Wilson A., Allen E., Thalanany M.M., Hibbert C.L., Truesdale A., Clemens F., Cooper N. (2009). Efficacy and economic assessment of conventional ventilatory support versus extracorporeal membrane oxygenation for severe adult respiratory failure (CESAR): A multicentre randomised controlled trial. Lancet.

[143] Masur J., Freeman C., Mohan S. (2020). A Double-Edged Sword: Neurologic Complications and Mortality in Extracorporeal Membrane Oxygenation Therapy for COVID-19–Related Severe Acute Respiratory Distress Syndrome at a Tertiary Care Center. Am. J. Neuroradiol..

[144] Garg R.K., Paliwal V.K., Malhotra H.S., Sharma P.K. (2021). Neuroimaging Patterns in Patients with COVID-19-Associated Neurological Complications: A Review. Neurol. India.

[145] Rapalino O., Pourvaziri A., Maher M., Jaramillo-Cardoso A., Edlow B., Conklin J., Huang S., Westover B., Romero J., Halpern E. (2021). Clinical, Imaging, and Lab Correlates of Severe COVID-19 Leukoencephalopathy. Am. J. Neuroradiol..

[146] Radmanesh A., Derman A., Lui Y.W., Raz E., Loh J.P., Hagiwara M., Borja M.J., Zan E., Fatterpekar G.M. (2020). COVID-19—Associated Diffuse Leukoencephalopathy and Microhemorrhages. Radiology.

[147] Soares M.N., Eggelbusch M., Naddaf E., Gerrits K.H.L., van der Schaaf M., van den Borst B., Wiersinga W.J., van Vugt M., Weijs P.J.M., Murray A.J. (2022). Skeletal muscle alterations in patients with acute Covid-19 and post-acute sequelae of Covid-19. J. Cachex. Sarcopenia Muscle.

[148] Schiaffino S., Albano D., Cozzi A., Messina C., Arioli R., Bnà C., Bruno A., Carbonaro L.A., Carriero A., Carriero S. (2021). CT-derived Chest Muscle Metrics for Outcome Prediction in Patients with COVID-19. Radiology.

[149] Levy D., Giannini M., Oulehri W., Riou M., Marcot C., Pizzimenti M., Debrut L., Charloux A., Geny B., Meyer A. (2022). Long Term Follow-Up of Sarcopenia and Malnutrition after Hospitalization for COVID-19 in Conventional or Intensive Care Units. Nutrients.

[150] Welch C., Greig C., Masud T., Wilson D., Jackson T.A. (2020). COVID-19 and Acute Sarcopenia. Aging Dis..

[151] Pironi L., Sasdelli A.S., Ravaioli F., Baracco B., Battaiola C., Bocedi G., Brodosi L., Leoni L., Mari G.A., Musio A. (2020). Malnutrition and nutritional therapy in patients with SARS-CoV-2 disease. Clin. Nutr..

[152] Cruz-Jentoft A.J., Sayer A.A. (2019). Sarcopenia. Lancet.

[153] Lee K., Shin Y., Huh J., Sung Y.S., Lee I.-S., Yoon K.-H., Kim K.W. (2019). Recent Issues on Body Composition Imaging for Sarcopenia Evaluation. Korean J. Radiol..

[154] Giraudo C., Librizzi G., Fichera G., Motta R., Balestro E., Calabrese F., Carretta G., Cattelan A.M., Navalesi P., Pelloso M. (2021). Reduced muscle mass as predictor of intensive care unit hospitalization in COVID-19 patients. PLoS ONE.

[155] Damanti S., Cristel G., Ramirez G.A., Bozzolo E.P., Da Prat V., Gobbi A., Centurioni C., Di Gaeta E., Del Prete A., Calabrò M.G. Influence of reduced muscle mass and quality on ventilator weaning and complications during intensive care unit stay in COVID-19 patients. Clin. Nutr..

[156] Ng M.-Y., Lee E.Y., Yang J., Yang F., Li X., Wang H., Lui M.M.-S., Lo C.S.-Y., Leung B., Khong P.-L. (2020). Imaging Profile of the COVID-19 Infection: Radiologic Findings and Literature Review. Radiol. Cardiothorac. Imaging.

[157] Rubin G.D., Ryerson C.J., Haramati L.B., Sverzellati N., Kanne J.P., Raoof S., Schluger N.W., Volpi A., Yim J.-J., Martin I.B. (2020). The Role of Chest Imaging in Patient Management during the COVID-19 Pandemic: A Multinational Consensus Statement from the Fleischner Society. Chest.

[158] Gandhi D., Jain N., Khanna K., Li S., Patel L., Gupta N. (2020). Current role of imaging in COVID-19 infection with recent recommendations of point of care ultrasound in the contagion: A narrative review. Ann. Transl. Med..

[159] Frija G., Blažić I., Frush D.P., Hierath M., Kawooya M., Donoso-Bach L., Brkljačić B. (2021). How to improve access to medical imaging in low- and middle-income countries?. EClinicalMedicine.

[160] Blažić I., Brkljačić B., Frija G. (2021). The use of imaging in COVID-19—results of a global survey by the International Society of Radiology. Eur. Radiol..

